# Percutaneous Cryoneurolysis for Upper Limb Spasticity: A Systematic Scoping Review of Current Evidence, Safety Profile, and Implications for Hand and Upper Extremity Practice

**DOI:** 10.3390/jcm15093541

**Published:** 2026-05-06

**Authors:** Marco Passiatore, Luciano Maria Bissolotti, Marta Starnoni, Luca Pecori, Anna Paola D’Apolito, Francesco De Santis, Rocco De Vitis

**Affiliations:** 1 Department of Medical and Surgical Specialties, Radiological Sciences, and Public Health, University of Brescia, Via Branze 38, 25123 Brescia, Italy; 2 Fondazione Teresa Camplani, Via Moretto 33, 25122 Brescia, Italy; luciano.bissolotti@fondazionecamplani.it; 3 Burn Center, Department of Plastic Surgery, Padua University Hospital, Via Nicolò Giustiniani 2, 35128 Padua, Italy; martastarn@gmail.com; 4 Fondazione Policlinico Universitario Agostino Gemelli IRCCS, Largo Agostino Gemelli 8, 00168 Rome, Italy; roccodevitis@yahoo.com

**Keywords:** muscle spasticity, cryotherapy, upper extremity, hand, nerve block, botulinum toxins, type A, neurotomy, neurological rehabilitation, GRADE

## Abstract

**Background/Objectives:** Percutaneous cryoneurolysis (CNL) has emerged as a minimally invasive neuromodulatory technique for focal spasticity management, with growing international clinical adoption since 2018. Its application to upper limb motor nerve targets—including branches of the musculocutaneous, radial, median, ulnar, pectoral, and thoracodorsal nerves—is of direct relevance to clinicians involved in the surgical and non-surgical management of hand and upper extremity spasticity. The existing literature lacks a comprehensive systematic appraisal of its evidence base. This systematic scoping review aimed to map all published evidence on CNL for spasticity across all aetiological groups and anatomical regions, with particular attention to upper limb and hand-relevant targets; appraise methodological quality using design-appropriate tools; characterise the safety profile; apply the Grading of Recommendations Assessment, Development and Evaluation (GRADE) framework to key outcome domains; and identify critical evidence gaps. **Methods:** A systematic scoping review was conducted following the Preferred Reporting Items for Systematic Reviews and Meta-Analyses extension for Scoping Reviews (PRISMA-ScR) guidelines (search through February 2026). PubMed/MEDLINE, Embase (via Ovid), Scopus, and the Cochrane Library were searched. Methodological quality was assessed using JBI Critical Appraisal Checklists, Risk Of Bias In Non-randomised Studies of Interventions (ROBINS-I), and A MeaSurement Tool to Assess systematic Reviews-2 (AMSTAR-2). Certainty of evidence was evaluated using GRADE. **Results:** Twenty-five studies met inclusion criteria; no randomised controlled trials (RCTs) were identified. In the largest prospective observational cohort (n = 59, 12-month follow-up), CNL produced statistically significant improvements in passive range of motion (ROM), Modified Ashworth Scale (MAS) scores, and pain in patients with upper limb spasticity refractory to botulinum toxin type A (BoNT-A). A prospective safety study (n = 113 patients; 277 nerves) documented that 96.75% of nerve treatments produced no post-procedural sensory disturbance; the risk was approximately 10-fold higher for mixed sensorimotor than purely motor nerve targets (7.1% vs. 1.1%). Certainty of evidence was Very Low (⊕◯◯◯) for all efficacy outcomes and Low (⊕⊕◯◯) for safety. **Conclusions:** CNL represents a mechanistically sound second-line or complementary intervention for refractory focal spasticity. In the upper extremity context, it may additionally serve as a reversible functional evaluation tool before irreversible surgical decisions—including selective neurotomy—are made. The evidence base is critically constrained by the absence of RCTs, confirmed cohort overlap between the two largest primary studies, financial conflicts of interest with the primary device manufacturer identified in ≥48% of included studies (≥12/25), and single-institution concentration of primary evidence (≥69% of primary clinical studies from one research group). Multiple ongoing controlled trials are expected to provide higher-quality evidence to inform guideline development.

## 1. Introduction

### 1.1. Spasticity: Definition and Clinical Burden

Spasticity, classically defined by Lance in 1980 as “a motor disorder characterised by a velocity-dependent increase in tonic stretch reflexes with exaggerated tendon jerks, resulting from hyperexcitability of the stretch reflex” [1], is one of the most prevalent and disabling manifestations of upper motor neuron lesions. Contemporary definitions have broadened this concept to encompass impairment of voluntary motor control, pathological co-contraction, and secondary changes in muscle biomechanical properties [2].

The global burden of spasticity is substantial. Post-stroke spasticity develops in 20–40% of survivors [3]; prevalences reach 60–90% in multiple sclerosis (MS) [4] and 65–78% in spinal cord injury (SCI) [5]. The clinical impact is multidimensional: spasticity causes chronic pain through muscle spasms and musculoskeletal overload, leads to secondary complications including fixed contractures and pressure ulcers [6], and significantly compromises quality of life across physical, psychological, and social domains [7]. The associated caregiver burden is substantial, and annual healthcare costs per patient range from several thousand to tens of thousands of euros [8].

In the upper limb, spasticity produces pathological flexor synergy patterns—involving shoulder adduction and internal rotation, elbow flexion, forearm pronation, wrist flexion, and finger and thumb flexion—that progressively impair hand function, hygiene, and independence in activities of daily living [7]. For hand and upper extremity specialists, these patterns represent a clinically challenging therapeutic target: the choice between reversible pharmacological interventions and irreversible surgical procedures, each with distinct risk–benefit profiles, constitutes one of the most consequential decisions in upper extremity spasticity management [7,8,9,10,11,12,13,14,15].

### 1.2. Pathophysiology

Spasticity arises from complex interactions between neural alterations and secondary muscle property changes. The primary neurological mechanism involves loss of descending inhibitory influences from supraspinal centres, leading to spinal circuit disinhibition and hyperexcitability of alpha and gamma motoneurons [1,2]. The stretch reflex becomes pathologically hyperactive, with a reduced activation threshold and amplified response, exacerbated by reduced presynaptic inhibition of Ia afferents and impaired reciprocal inhibition between antagonist muscle groups [9].

Maladaptive plasticity of spinal circuits—including axonal sprouting, synaptic modifications, and altered neurotransmitter balance—consolidates pathological patterns perpetuating spasticity over time [2]. At the muscle level, chronic spasticity induces structural changes: reduced sarcomere numbers, increased connective tissue with collagen deposition, and altered contractile properties, which underlie the “fixed” component of resistance distinguishable from the velocity-dependent “dynamic” component [10]. These structural muscle changes are clinically relevant in the upper extremity context, as they determine the degree to which tone reduction—achieved pharmacologically or through peripheral nerve intervention—can translate into functional hand gain in the absence of concurrent orthopaedic procedures.

### 1.3. Current Therapeutic Landscape and Limitations

Current management combines pharmacological, physical, and surgical approaches, each with significant limitations. Oral antispastic medications (baclofen, tizanidine, benzodiazepines, dantrolene) reduce spasticity but cause dose-dependent central adverse effects—sedation, weakness, and cognitive impairment—and lack anatomical selectivity, potentially compromising residual voluntary function [11].

Botulinum toxin type A (BoNT-A) represents the gold standard for focal spasticity, supported by Level A evidence and endorsed by international consensus guidelines [12,16]. However, three structural limitations constrain its long-term utility: (i) the median efficacy window is only 12–16 weeks, necessitating repeated treatment cycles [12]; (ii) regulatory dose ceilings (400 U OnabotulinumtoxinA per 12 weeks in most jurisdictions) may be insufficient for severe or distributed spasticity patterns [17]; and (iii) progressive tachyphylaxis secondary to neutralising antibody development limits long-term responsiveness in a clinically relevant proportion of patients [16].

Chemical neurolysis with phenol or alcohol can extend tone reduction for 3–6 months but carries meaningful risks of painful dysaesthesia in 10–30% of cases and difficult-to-manage chemical neuritis [18]. Intrathecal baclofen (ITB) is effective for severe generalised spasticity but requires neurosurgical implantation, life-long specialised follow-up, and is associated with serious complications including pump malfunction and catheter-related infection [19]. Surgical options such as selective dorsal rhizotomy and selective neurotomy are irreversible, carry procedure-related risks, and are appropriate only for carefully selected candidates [20].

Selective surgical neurotomy, in particular, is a well-established technique in upper limb spasticity management: it achieves greater and more sustained tone reduction than BoNT-A [20] but is irreversible and, once performed, precludes functional re-evaluation under different tone conditions. For the hand and upper extremity surgeon, this irreversibility represents a clinically significant constraint: optimal timing and candidate selection require a reliable method of predicting functional outcome under simulated low-tone conditions, prior to committing to a permanent structural intervention.

### 1.4. The Therapeutic Gap and Rationale for Cryoneurolysis

Current treatment options present a clinically relevant dichotomy: focal interventions such as BoNT-A are effective but temporary with frequent re-administration requirements, while surgical options produce more durable effects at the cost of irreversibility and invasiveness. This leaves a substantial gap for an intermediate approach combining focality and reversibility with prolonged duration.

Patients who fail to respond to BoNT-A, who require doses exceeding approved ceilings, or who develop tachyphylaxis have limited evidence-based alternatives. The 3–4-month BoNT-A cycle imposes considerable patient and healthcare system burden with significant economic implications. Extending treatment intervals while maintaining clinical benefit would meaningfully improve quality of life and resource allocation.

Percutaneous cryoneurolysis (CNL) offers a mechanistically distinct alternative. CNL has been utilised for decades in chronic pain management [21]; its application to motor nerve targets for spasticity was first reported by Kim and Ferrante in 1998 [22] and was systematically operationalised by Winston and colleagues from 2018 onwards [23]. CNL uses extreme cold to produce a reversible Sunderland Grade II nerve lesion (axonotmesis) with preserved endoneurium, perineurium, and epineurium, permitting orderly axonal regrowth at approximately 1–2 mm per day [24]. This mechanism is fundamentally distinct from phenol/alcohol neurolysis—which induces irreversible neurotmesis with neuroma risk—and from BoNT-A—which acts at the neuromuscular junction without structural nerve disruption.

From the perspective of hand and upper extremity surgery, the reversibility of CNL holds a specific clinical interest: the temporary peripheral nerve silencing produced by axonotmesis allows functional assessment during the period of tone reduction, providing the multidisciplinary team—including the hand surgeon—with pre-decisional information analogous to, but of substantially longer duration than, a diagnostic nerve block (DNB). This potential role as a reversible pre-surgical functional evaluation tool—capable of informing the timing and candidate selection for selective neurotomy and other irreversible procedures—deserves prospective investigation in dedicated upper extremity surgery cohorts.

It must be emphasised that mechanistic plausibility, however scientifically compelling, does not constitute clinical evidence of efficacy and is not invoked in this review as a substitute for controlled trial data.

### 1.5. Objectives

The primary research question of this systematic scoping review, formulated according to a modified PICO framework adapted for scoping reviews, is as follows:

**Population (P):** Adult and paediatric patients of any age with spasticity of any aetiology (post-stroke, multiple sclerosis, spinal cord injury, cerebral palsy, traumatic brain injury, and other upper motor neuron syndrome aetiologies), with particular attention to upper limb and hand-relevant presentations.

**Intervention (I):** Percutaneous cryoneurolysis (CNL) of peripheral motor, sensorimotor, or mixed nerves, performed under image guidance (ultrasound with or without electrical nerve stimulation), using any cryogenic device and any treatment parameter configuration.

**Comparator (C):** Any comparator (botulinum toxin type A, chemical neurolysis, selective surgical neurotomy, physical therapy, sham/no treatment) or no comparator, consistent with scoping review methodology.

**Outcomes (O):** Spasticity scales (Modified Ashworth Scale [MAS], Modified Tardieu Scale [MTS]); passive and active range of motion (ROM); pain scores; functional assessments (Goal Attainment Scaling [GAS], Disabilities of the Arm, Shoulder and Hand [DASH] questionnaire, ArmA/LegA scales); quality of life; adverse events; cost-effectiveness.

**Study designs (S):** All study designs (RCTs, non-randomised comparative studies, cohort studies, case series, case reports, systematic and scoping reviews, narrative reviews, and technical notes), consistent with the scoping review framework. This systematic scoping review aims to: (1) map the entirety of published evidence on CNL for spasticity across all aetiological groups and anatomical regions, with particular attention to upper limb and hand-relevant targets; (2) appraise the methodological quality and risk of bias of included studies using design-appropriate tools; (3) characterise the safety profile based on structured adverse-event data; (4) apply the GRADE framework to classify the certainty of evidence for key outcome domains; (5) identify critical evidence gaps and prioritise future research directions; and (6) contextualise the available evidence within the clinical decision-making framework relevant to hand and upper extremity surgery specialists.

## 2. Materials and Methods

### 2.1. Study Design and Reporting

This study was conducted as a systematic scoping review following the methodological framework of Arksey and O’Malley and Levac et al. [25,26], and is reported in accordance with the PRISMA-ScR guidelines [27].

A systematic scoping review was identified as the methodologically appropriate design for this literature: scoping reviews are specifically indicated when the evidence base is heterogeneous in study design, predominantly of low methodological quality, and characterised by multiple critical evidence gaps requiring systematic mapping prior to the commissioning of higher-quality primary research [25,26]. The purpose of this review is therefore to map and appraise the existing evidence—not to confirm efficacy—consistent with the scoping review mandate.

The PRISMA 2020 flow diagram is provided in Figure 1 [28]. No prospective protocol registration was performed; this constitutes a limitation acknowledged in Section 4.5. PRISMA-ScR Checklist is reported as (Supplementary Materials Table S1).

### 2.2. Search Strategy

A comprehensive literature search was conducted across multiple electronic databases from January 2005 to 25 February 2026. Primary databases included PubMed/MEDLINE, Embase (via Ovid), and the Cochrane Library. Secondary databases included Scopus, CINAHL, PEDro, and ClinicalTrials.gov. Grey literature sources included Google Scholar (first 200 citations) and conference proceedings (American Academy for Cerebral Palsy and Developmental Medicine [AACPDM], American Society of Neurorehabilitation [ASNR], American Academy of Physical Medicine and Rehabilitation [AAPM&R]).

The search strategy was developed following a modified Participants, Intervention, Comparator, Outcomes (PICO) framework adapted for intervention studies. For PubMed/MEDLINE, the following search strings were constructed:

**#1 Intervention—Cryoneurolysis:**

(“cryoneurolysis” OR “cryo-neurolysis” OR “cryoablation” OR “cryo-ablation” OR “cryoanalgesia” OR “cryotherapy” [MeSH] OR “nerve cryoablation” OR “percutaneous cryoneurolysis” OR “ultrasound-guided cryoablation” OR “cryogenic neurolysis”)

**#2 Condition—Spasticity:**

(“spasticity” [MeSH] OR “spasticity” OR “spastic” OR “muscle spasticity” OR “hypertonia” OR “hypertonicity” OR “upper motor neuron syndrome” OR “spastic paresis” OR “spastic paralysis”)

**Final combination:** #1 AND #2

Filters applied initially included publication dates from January 2005 to February 2026, and language restrictions to English, Italian, French, Spanish, and German. No study design filters were applied at the initial search stage to maximise sensitivity. The seminal pre-2005 case report by Kim and Ferrante (1998) [22] was recovered through backward citation chasing and included as an exception for its unique historical contribution.

The search strategy was adapted for Embase (via Ovid) using Emtree controlled vocabulary as follows:

**#1 Intervention—Cryoneurolysis (Embase/Ovid):**

(exp cryotherapy/OR cryoneurolysis.mp. OR cryo-neurolysis.mp. OR cryoablation.mp. OR cryo-ablation.mp. OR cryoanalgesia.mp. OR “nerve cryoablation”.mp. OR “percutaneous cryoneurolysis”.mp. OR “ultrasound-guided cryoablation”.mp. OR “cryogenic neurolysis”.mp.)

**#2 Condition—Spasticity (Embase/Ovid):**

(exp spasticity/OR exp muscle spasm/OR spasticity.mp. OR spastic.mp. OR “muscle spasticity”.mp. OR hypertonia.mp. OR hypertonicity.mp. OR “upper motor neuron syndrome”.mp. OR “spastic paresis”.mp. OR “spastic paralysis”.mp.)

**Final combination:** #1 AND #2

For the Cochrane Library, the search strategy was constructed using MeSH descriptors with free-text expansion as follows:

**#1 Intervention—Cryoneurolysis (Cochrane Library):**

[mh “Cryotherapy”] OR (cryoneurolysis OR “cryo-neurolysis” OR cryoablation OR “cryo-ablation” OR cryoanalgesia OR “nerve cryoablation” OR “percutaneous cryoneurolysis” OR “ultrasound-guided cryoablation” OR “cryogenic neurolysis”) in Title, Abstract, Keywords

**#2 Condition—Spasticity (Cochrane Library):**

[mh “Muscle Spasticity”] OR (spasticity OR spastic OR “muscle spasticity” OR hypertonia OR hypertonicity OR “upper motor neuron syndrome” OR “spastic paresis” OR “spastic paralysis”) in Title, Abstract, Keywords

**Final combination:** #1 AND #2

All search strings were executed without study design filters at the initial stage to maximise sensitivity, and were independently verified by both reviewers prior to execution.

### 2.3. Complementary Search Methods

To ensure comprehensive coverage, four complementary methods were employed: backward citation chasing of reference lists of all included studies and relevant reviews; forward citation tracking and electronic PubMed alerts maintained throughout the review period; expert author searching of the principal investigators of the Canadian Advances in Neuro-Orthopedics for Spasticity Consortium (CANOSC) group; and hand searching of targeted journals (PM&R, Archives of Physical Medicine and Rehabilitation, American Journal of Physical Medicine & Rehabilitation, Pain Medicine, Developmental Medicine & Child Neurology) for the preceding five years.

### 2.4. Inclusion and Exclusion Criteria

**Inclusion criteria:** (1) studies investigating percutaneous CNL of peripheral nerves for the treatment of spasticity of any aetiology; (2) human subjects of any age; (3) any study design (RCTs, non-randomised comparative studies, cohort studies, case series, case reports, systematic and scoping reviews); (4) studies reporting clinical outcomes (spasticity scales, range of motion [ROM], functional assessments, pain scores, quality of life, or adverse events); (5) studies describing technical aspects or mechanisms of action of CNL relevant to spasticity treatment, with particular attention to upper limb and hand-relevant anatomical targets and outcome measures.

**Exclusion criteria:** (1) studies on superficial cryotherapy (ice packs, cold baths, cooling garments) without nerve-targeted cryoablation; (2) whole-body cryotherapy or cryogenic chambers; (3) cryoablation for oncological purposes; (4) studies exclusively in animal models, unless essential for mechanistic understanding; (5) conference abstracts without sufficient methodological detail; (6) editorials, commentaries, or letters without original data; (7) full texts unavailable after institutional access and author contact attempts.

### 2.5. Study Selection

All records were imported into reference management software (Mendeley Reference Manager v2.144.0 Zotero 7.0), and duplicates were removed by automated and manual methods. Two independent reviewers screened titles and abstracts against eligibility criteria, applying liberal inclusion criteria at this stage. Full-text articles were independently assessed by both reviewers; disagreements were resolved by discussion or third-party adjudication. Reasons for full-text exclusion were documented in the PRISMA flow diagram (Figure 1 [28]).

### 2.6. Data Extraction

A standardised data extraction form was developed and pilot-tested. One reviewer extracted data from all included studies; a second reviewer verified accuracy for a random 20% sample. Extracted items included: study characteristics (first author, year, country, design); patient characteristics (age, sex, diagnosis, spasticity distribution, baseline severity); intervention details (target nerves, device, treatment parameters, guidance technique, diagnostic nerve block use, number of sessions); outcome measures and timing; safety data (incidence, type, severity, management of adverse events); and concomitant interventions.

### 2.7. Quality Assessment

Methodological quality was assessed using design-specific tools following Joanna Briggs Institute (JBI) Manual for Evidence Synthesis guidance [29] (Table 1).

Quality assessment was performed independently by two reviewers; disagreements were resolved by consensus or third-party adjudication.

**Methodological note on ROBINS-I:** ROBINS-I was originally developed and validated for comparative effectiveness studies with at least two comparison groups [31]. Its application to single-arm before-after designs—as in both observational studies included here—requires domain-specific adaptation, as noted by Sterne et al. [31]. The following adaptations were applied: D1 (Confounding) was rated Critical in the absence of a comparator arm, precluding control for spontaneous disease trajectory, regression to the mean, and natural history effects; D2 (Selection of participants) was assessed on the basis of selection pathway and institutional representativeness rather than differential selection between treatment groups; D4 (Deviations from intended interventions) was adapted to assess within-arm protocol adherence rather than differential co-intervention between groups; D5 (Missing data) was applied to within-group attrition and incomplete follow-up rather than differential dropout between arms; D6 (Measurement of outcomes) and D7 (Selection of reported results) were applied without modification, focusing on measurement bias and selective outcome reporting within the single cohort. This adaptation is explicitly acknowledged as a methodological limitation of the present review (Section 4.5).

### 2.8. Evidence Synthesis and GRADE Assessment

Given the anticipated heterogeneity in patient populations, interventions, comparators, outcomes, and study designs, a narrative synthesis approach was employed rather than quantitative meta-analysis. The overall certainty of evidence for key outcome domains was assessed using the GRADE framework [33].

The GRADE framework was applied to the following pre-specified outcome domains: (1) spasticity reduction (MAS); (2) range of motion improvement (Tardieu V1, goniometry); (3) pain reduction (VAS/NRS); (4) safety/adverse events (sensory disturbance, local complications, serious adverse events); (5) functional outcomes (GAS, ArmA/LegA scales, DASH questionnaire); (6) quality of life and cost-effectiveness.

The following downgrading criteria were applied across all outcome domains:

Risk of bias: Downgraded two levels for all efficacy outcomes, reflecting the Critical-to-Serious overall ROBINS-I ratings of the two largest primary clinical studies; the complete absence of a comparator arm in all included efficacy studies; the absence of assessor blinding in all included studies (the treating clinician serving as outcome assessor in multiple visits); and financial conflicts of interest with the primary device manufacturer identified in ≥48% of included studies (≥12/25); and systematic outcome assessment without assessor blinding.

Indirectness: Downgraded one level for all efficacy outcomes, reflecting the single-arm uncontrolled design of all primary efficacy studies and the use of the Modified Ashworth Scale (MAS) as primary outcome—an ordinal measure with well-documented psychometric limitations that does not constitute a validated patient-relevant functional endpoint.

Imprecision: Downgraded one level for all efficacy outcomes, reflecting the small sample size of the largest efficacy study (n = 59), the absence of confidence intervals for the majority of reported outcome estimates, and the inability to perform quantitative pooling across heterogeneous studies.

Publication bias: Downgraded one level for all efficacy outcomes, reflecting the near-exclusive origin of positive results from a single research institution and the absence of published null or negative outcome studies in this literature.

Safety outcomes: Downgraded one level rather than two, reflecting the partial availability of structured prospective adverse-event data from a dedicated safety study (Winston et al. 2023 [34], n = 113 patients, 277 nerves treated) with pre-specified adverse-event collection, yielding a certainty rating of Low (⊕⊕◯◯) rather than Very Low. The remaining downgrade reflects the absence of standardised adverse-event collection frameworks (e.g., Common Terminology Criteria for Adverse Events [CTCAE]) across the majority of included studies and the unsystematically documented long-term safety profile beyond 12 months.

## 3. Results

### 3.1. Search Results and Study Selection

The systematic database search identified 220 total records: 93 from PubMed/MEDLINE, 88 from Embase (via Ovid MEDLINE), and 39 from Scopus. Following automated and manual deduplication (estimated 76 records removed, primarily reflecting overlap between PubMed and Embase/Ovid MEDLINE), 144 unique records were advanced to title and abstract screening.

Ninety-seven records were excluded at this stage. The most frequent reasons for exclusion were: absence of any relationship to cryoneurolysis or spasticity (n = 22), neonatal therapeutic hypothermia studies unrelated to the intervention (n = 14), superficial cryotherapy without targeted nerve ablation (n = 12), oncological cryoablation (n = 11), spinal cord protective hypothermia during cardiovascular surgery (n = 9), and irrelevant animal models (n = 7). The remaining 47 records were retrieved for full-text assessment.

Complementary search strategies identified 13 additional records: 8 through backward citation chasing, 3 through alert systems and forward citation tracking, 1 through expert author searching of CANOSC principal investigators, and 1 through hand searching of targeted journals. The seminal 1998 case report by Kim and Ferrante [22] was recovered through backward citation chasing, having been excluded by the initial date filter. Two articles published in January 2026 (Chantraine et al. [35]; Di Lorenzo et al. [36]) were captured through the alert system.

A total of 60 full-text articles were formally assessed for eligibility. Thirty-five were excluded with documented reasons (Figure 1—PRISMA Flow Diagram [28]).

**Twenty-five studies** met all inclusion criteria and were included in the qualitative synthesis. The final corpus comprised 10 case reports, 3 case series, 2 prospective observational studies, 3 technical notes/video gallery publications, 4 narrative or expert reviews, 2 systematic or scoping reviews, and 1 device technology review. No RCTs were identified. Quantitative meta-analysis was not performed.

**A critical finding** concerns the pervasive conflict of interest landscape across the included literature. The majority of clinical studies (≥12/25) involve Winston P as principal or co-investigator, who has consistently declared educational grants, research grants, honoraria, and advisory board membership for Pacira BioSciences, Inc. (Swindon, UK)—the manufacturer of the iovera° device used in the majority of included studies—as well as AbbVie (North Chicago, IL, USA), Ipsen (Paris, France), and Merz (Frankfurt am Main, Germany). The largest observational study (Hashemi et al. 2025 [37], n = 59) was directly funded by Pacira BioSciences and AbbVie, included a Pacira employee with stock ownership as co-author, and had its writing and editorial assistance funded by Pacira. Guynn (2025) [38] is a declared paid consultant of Pacira. Pick et al. (2025) [39] received in-kind device support from Pacira BioSciences UK. These findings represent a substantial sponsorship bias risk that must be explicitly considered when interpreting the evidence base.

Financial conflicts of interest involving the primary device manufacturer (Pacira BioSciences, Inc.) were identified in ≥12 of 25 included studies (48%), including the two largest primary clinical studies. A single research group (CANOSC/Vancouver Island Health Authority) is responsible for ≥11 of 16 primary clinical studies (69%), constituting a marked single-institution concentration of primary evidence.

### 3.2. Characteristics of Included Studies

#### 3.2.1. Overview

The included studies span nearly three decades, from the seminal 1998 case report by Kim and Ferrante [22] to two studies published in January 2026 [35,36]. The majority of primary clinical studies (16/25) were published between 2023 and 2026, reflecting the rapid growth of this field following broader clinical adoption of the percutaneous CNL approach. The complete list of included studies by category is provided in Table 2.

#### 3.2.2. Geographic Distribution and Research Groups

A striking feature of the included literature is marked geographic and institutional concentration. The large majority of primary clinical studies originate from a single academic institution: Vancouver Island Health Authority (VIHA)/University of British Columbia (Victoria, BC, Canada), under the leadership of Winston P and collaborators of the CANOSC group. Winston P appears as principal investigator or co-author in at least 12 of the 25 included studies. The remaining primary clinical studies were conducted in the United Kingdom (Pick et al. 2025 [39]), the United States (Guynn 2025 [38]; Kim & Ferrante 1998 [22]), Luxembourg (Chantraine et al. 2026 [35]), and Italy (Di Lorenzo et al. 2026 [36]). This concentration of primary clinical evidence within a single research group constitutes a significant limitation of the current evidence base.

#### 3.2.3. Patient Populations

The two largest primary clinical studies are:

Hashemi et al. 2025 [37]: a repeated-measures, single-centre, observational pilot study (NCT04670783) in 59 adults with upper limb spasticity refractory to or plateaued on botulinum therapies. Mean age 59.5 (SD 16.2) years; 52.5% female (n = 31); 45.8% male (n = 27). Underlying diagnoses: cerebrovascular accident/stroke (n = 49), spinal cord injury (n = 3), cerebral palsy (CP) (n = 2), multiple sclerosis (n = 2), traumatic brain injury (TBI) (n = 2).

Risk of bias (ROBINS-I): Overall rating—Critical. Domain-level ratings: Confounding (Critical): no comparator arm; no adjustment for BoNT-A wash-out period or concomitant rehabilitation; spasticity severity at baseline not stratified. Selection bias (Serious): single-institution convenience sample; no documentation of consecutive enrolment or formal eligibility screening log. Classification of intervention (Low): ultrasound-guided procedure with standardised device and documented treatment parameters. Deviations from intended intervention (Low): no cross-over or co-intervention documented. Missing outcome data (Moderate): 12-month completion rate not explicitly reported per individual outcome domain. Outcome measurement (Critical): treating clinician served as outcome assessor at all time points; no assessor blinding; MAS is an ordinal scale with documented interrater variability. Selection of reported results (Serious): trial registration (NCT04670783) predated study completion; alignment between pre-registered and reported outcomes not formally verified.

Winston et al. 2023 [34]: a prospective safety study in which CNL was performed on 277 nerves (99 mixed motor–sensory) in 113 patients (59 female, 54 male, mean age 54.4 years), prospectively recruited across three cohort studies at a single institution.

Risk of bias (ROBINS-I): Overall rating—Serious. Domain-level ratings: Confounding (Moderate): safety outcome (sensory adverse events) is less susceptible to confounding than efficacy outcomes; however, no adjustment for pre-existing neuropathy, diabetes mellitus, or prior neurolysis procedures was documented. Selection bias (Serious): single-institution convenience sample pooled across three heterogeneous cohort studies with differing anatomical targets; no documentation of consecutive enrolment. Classification of intervention (Low): standardised ultrasound-guided procedure with documented nerve-level treatment parameters. Deviations from intended intervention (Low). Missing outcome data (Moderate): adverse event follow-up duration varied across the three constituent cohorts; standardised follow-up intervals not uniformly applied. Outcome measurement (Moderate): adverse events prospectively collected; however, no standardised classification framework (e.g., Common Terminology Criteria for Adverse Events [CTCAE] or MedDRA) was applied; severity grading was not pre-specified. Selection of reported results (Moderate).

Critical methodological note—cohort overlap: The 59 participants of Hashemi et al. 2025 (NCT04670783) [37] constitute a formally confirmed subset of the 113 patients of Winston et al. 2023 [34]. Winston et al. [34] explicitly states that 59 individuals were enrolled in the upper limb study, 43 in the tibial nerve study, and 11 in the bilateral obturator study. These are therefore not independent samples, and double-counting in evidence interpretation must be explicitly avoided. The net non-overlapping patient contribution of Winston et al. 2023 [34] to the aggregate clinical sample is therefore 54 individuals (43 tibial nerve cohort + 11 bilateral obturator cohort). Efficacy data from Hashemi et al. 2025 [37] and safety data from Winston et al. 2023 [34] cannot be combined additively for any outcome domain without explicit correction for this overlap.

Combining all primary clinical studies and accounting for confirmed cohort overlap, the net aggregated independent primary clinical sample is estimated at 155–170 individual patients (upper bound 200–215 prior to overlap correction). Aetiology of spasticity was heterogeneous across included clinical studies, comprising post-stroke, multiple sclerosis, cerebral palsy, spinal cord injury, traumatic brain injury, and neurodegenerative tetraparesis. Age range extended from paediatric patients (minimum 14 years [40]) to older adults (range 25–75 years documented in Pick et al. 2025 [39]). The limited sample size, combined with the single-institution concentration of evidence and the confirmed inter-study cohort overlap, constitutes a major source of imprecision and selection bias that informed the GRADE downgrading applied to all outcome domains (Section 2.8 and Section 3.3.6). Patient Cohort Independence Status and Registry Cross-Reference across primary clinical studies are reported in Table 2.

#### 3.2.4. Anatomical Targets and Device Characteristics

CNL procedures targeted a broad spectrum of predominantly motor, mixed sensorimotor, and purely motor nerve trunks and branches across both upper and lower limbs. In the largest observational study, upper limb targets included nerves supplying shoulder (n = 47), elbow (n = 33), wrist (n = 18), and fingers/thumb (n = 29) muscles [37]. This comprehensive upper limb coverage—encompassing the principal motor nerve branches controlling shoulder adduction and internal rotation, elbow flexion, forearm pronation, wrist flexion, and finger and thumb flexion—corresponds directly to the anatomical territories addressed in surgical upper extremity spasticity management. Lower limb targets across included studies included tibial nerve branches (soleus, gastrocnemius medialis and lateralis), femoral nerve branches (rectus femoris, vastus intermedius), obturator nerve (anterior and posterior divisions), sciatic nerve intramuscular branches (hamstrings), and the superficial fibular nerve.

**Guidance technique:** Ultrasound guidance combined with electrical nerve stimulation was the predominant approach across all included clinical studies. The diagnostic nerve block (DNB) protocol—using local anaesthetic blocks to pre-screen targets prior to definitive CNL—was systematically employed by the CANOSC group and adopted in Pick et al. 2025 [39].

**Device heterogeneity:** Although the iovera° system (Pacira BioSciences, Inc., Parsippany, NJ, USA; N_2_O cryogen, −88 °C) was the predominant device across primary clinical studies from the CANOSC group, it was not the sole device used across the corpus (Table 3):

This device heterogeneity—spanning three manufacturers and at least four distinct cryogen/temperature configurations—partially attenuates the device-manufacturer bias concern for studies using non-Pacira equipment and introduces an additional source of technical heterogeneity across the evidence base. Importantly, studies using alternative devices (Chantraine 2026 [35], Di Lorenzo 2026 [36], Scobie 2021 [41]) also declared absence of financial conflicts of interest with Pacira BioSciences.

#### 3.2.5. Outcome Measures

Outcome measurement was highly heterogeneous across included studies, reflecting the absence of a standardised outcome battery in the field. The MAS was reported in 13/16 primary clinical studies and used universally as the primary spasticity outcome. The Modified Tardieu Scale (MTS) was reported in 6/16 primary clinical studies. Goniometry-based passive and active ROM was reported in 14/16 primary clinical studies. Functional assessments included: Goal Attainment Scaling (GAS) and ArmA/LegA scales [39]; Patient-Reported Impact of Spasticity Measure (PRISM) [39]; quantitative gait analysis (Gait Deviation Index [GDI], Gillette Gait Index) and fine-wire electromyography (EMG) [35]; and caregiver-reported outcomes using the Care and Comfort Caregiver Questionnaire (CareQ) [41]. Quality of life and cost-effectiveness assessment was formally reported in one case report only [42]. Among upper limb-specific instruments, the Disabilities of the Arm, Shoulder and Hand (DASH) questionnaire was reported in Hashemi et al. 2025 [37], representing the most clinically pertinent functional measure for hand and upper extremity specialists among those employed in the included studies.

#### 3.2.6. Follow-Up Duration

Follow-up duration varied substantially across study types (Table 4).

The longest individual follow-up reported was 15 months (Boissonnault et al. 2024 [47]), with re-treatment performed at the end of the initial treatment window.

### 3.3. Quality Assessment and Risk of Bias

#### 3.3.1. Case Reports (JBI 8-Item Checklist)

Summary findings across the 10 case reports are presented in Table 3.

**Strengths consistently observed:** All 10 case reports provided detailed descriptions of the CNL technique, guidance modality, nerve targets, and probe parameters (Item 5: Intervention—Y in all 10). Post-intervention clinical condition was clearly reported in 9/10 cases (Item 6). All 10 case reports included a discussion of generalisable lessons (Item 8).

**Weaknesses consistently observed:** Demographic data were incomplete in 6/10 case reports; race/ethnicity was absent in all 10 (Item 1: U or N in 6 studies). Structured chronological timelines were provided in only 4/10 studies (Item 2). Adverse event reporting was explicit only in 4/10 studies; in the remaining six, absence of adverse events was implied but not formally documented (Item 7).

Three studies achieved **High** overall quality (Hughes et al. 2025 [42]; Mumby et al. 2025 [40]; MacRae et al. 2023 [46]). Two achieved **Moderate** quality (Boissonnault et al. 2024 [47]; Herzog et al. 2023 [43]). Five were rated **Low** (Schatz et al. 2024 [45]; David et al. 2024 [44]; Scobie & Winston 2021 [41]; Kim & Ferrante 1998 [22]) or **Low–Moderate** (Chantraine et al. 2026 [35]).The quality of the studies is resumed in Table 5.

#### 3.3.2. Case Series (JBI 10-Item Checklist)

Pick et al. 2025 [39] achieved the highest quality among case series (**Moderate**). Inclusion criteria were pre-specified; demographic data were reported; validated outcome instruments were used (MAS, ArmA, LegA, GAS, PRISM); follow-up was structured and adequately long (9–12 months). Critical weaknesses included the absence of a statistical analysis plan and small sample size (n = 8). In-kind device and supply support from Pacira BioSciences UK represents a potential influence on reporting.

Winston et al. 2019 [23] was rated **Low** quality. As the seminal publication proposing the Canadian protocol, it has high historical importance; however, by current JBI standards it scored poorly on consecutive inclusion, completeness reporting, demographic reporting, and statistical analysis.

Guynn 2025 [38] received the lowest rating (**Low–Critically Low**). Inclusion criteria, consecutive enrolment, and completeness of the series were not documented. Manuscript editing and submission support were provided by MedThinkSciCom, funded by Pacira BioSciences—a recognised form of industry-sponsored publication support constituting a significant conflict of interest even without direct research funding. The author is additionally listed as a paid Pacira consultant. Given this overall risk profile, data from this study are not used to support efficacy claims in this review. Quality assessment of case series is resumed in Table 6.

#### 3.3.3. Risk of Bias in Observational Studies (ROBINS-I)

**Domain-specific rationale:**

**D1—Confounding (Critical for Hashemi 2025** [37]; **Serious for Winston 2023** [34]): Both studies lack a comparator group. In Hashemi et al., participants were selected for refractory/plateaued spasticity—a population with an unknown natural history trajectory that may include spontaneous tone fluctuation. The absence of a sham or wait-list control means regression to the mean, placebo effects, and physiotherapist attention effects cannot be excluded.Quality assessment of observational studies is resumed in Table 7.

**D2—Selection of participants (Serious, both studies):** Both studies enrolled patients at a single institution (VIHA/University of British Columbia) through a single referral pathway (CANOSC programme), introducing significant selection bias and limiting external validity.

**D3—Classification of interventions (Low, both studies):** Both studies used a consistent, protocol-defined CNL technique with ultrasound and nerve stimulator guidance and pre-procedural DNB screening.

**D6—Measurement of outcomes (Serious, both studies):** MAS, used as the primary spasticity outcome, has well-documented psychometric limitations including moderate interrater reliability and ordinal scaling. No study reported assessor blinding; the treating clinician was also the outcome assessor in multiple visits.

**D7—Selection of reported results (Critical for Hashemi 2025** [37]): The study was funded by Pacira BioSciences and AbbVie; a Pacira employee with stock ownership appears in the author list; and the sponsorship agreement explicitly states that sponsors may review manuscripts before submission. This pre-submission review clause, even if not exercised, constitutes a structural conflict of interest not adequately mitigated by post hoc declarations.

#### 3.3.4. Secondary Studies (AMSTAR-2)

Both included systematic/scoping reviews (Muradia & Prasad 2026 [48]; Di Lorenzo et al. 2026 [36]) were assessed as **Low to Critically Low confidence** by AMSTAR-2 criteria, primarily due to: absence of pre-registered protocols (a critical AMSTAR-2 item); incompletely described or narrow search strategies; absence of formal risk of bias assessment of included primary studies; and no assessment of publication bias.

Narrative reviews (n = 4), technical notes (n = 3), and the device review (n = 1) were not subjected to quantitative quality scoring, consistent with JBI guidance for textual evidence. These publications were included for contextual and mechanistic information only and were not used to support efficacy or safety claims.

#### 3.3.5. Cross-Cutting Risk of Bias Concerns

Beyond study-level assessments, five structural sources of bias affect the CNL for spasticity literature as a whole:

**Pervasive industry funding and financial conflicts of interest:** Pacira BioSciences, Inc. provided direct research funding, equipment funding, and/or manuscript preparation support to the majority of included clinical studies. This pattern of financial entanglement between evidence producers and the primary commercial stakeholder represents a critical structural conflict not adequately mitigated by disclosure alone.

**Single-group, single-institution concentration:** The overwhelming majority of primary clinical evidence originates from a single research group. Results may not be reproducible in other settings or by operators with different training; potential publication of overlapping patient datasets across multiple studies inflates the apparent breadth of the evidence base.

**Absence of randomisation, blinding, and control conditions:** No RCT or sham-controlled trial has been published. All efficacy evidence derives from uncontrolled before-after designs or single-case observations, precluding causal attribution.

**Outcome assessment bias:** MAS was universally used without assessor blinding; in several case reports, the treating clinician also assessed outcomes, introducing performance and detection bias.

**Publication bias and reporting selectivity:** The literature is composed almost entirely of positive case reports and case series from a single institution; negative or equivocal outcomes may not have been published.

#### 3.3.6. GRADE Summary

GRADE Evidence Profile are resumed in Table 8.

### 3.4. Results of Individual Studies

#### 3.4.1. Preliminary Note on Evidence Interpretation

The following sections describe results as reported by their respective authors. These results **cannot be interpreted as reliable estimates of the causal effect of CNL on spasticity** in the absence of a comparator group, blinded outcome assessment, and adequate control for confounding variables. The described improvements reflect pre-post changes not exclusively attributable to treatment.

#### 3.4.2. Prospective Observational Studies

**Hashemi et al. 2025** [37] (ROBINS-I overall: Critical)

In this repeated-measures, single-centre pilot study (NCT04670783), percutaneous CNL was applied to upper limb nerves and intramuscular branches of 59 adults with upper limb spasticity refractory to or plateaued on botulinum therapies. Overall, 47 participants received CNL targeting shoulder-innervating nerves, 33 for the elbow, 18 for the wrist, and 29 for the fingers/thumb; a total of 182 nerves were treated; more than half of participants (n = 32) received CNL in more than one region. At 12 months, statistically significant improvements from baseline were documented in V1 (maximal passive ROM), active ROM, and MAS score for shoulder flexion and abduction (*p* ≤ 0.001 for all V1 improvements), and in V1 and MAS score for shoulder external rotation. Similar results were observed for elbow extension V1 (mean change from baseline: +12.9° [SD 18.2°]; *p* = 0.007), active ROM (+21.3° [SD 27.0°]; *p* = 0.007), and MAS score (*p* ≤ 0.008 for all time points). Wrist extension MAS score was significantly improved at 3 and 12 months. Average daily pain was significantly reduced at 1, 3, 6, and 12 months (*p* ≤ 0.04 for all). Goal Attainment Scaling (GAS) showed a mean increase of 11.9 (SD 9.8) points at 12 months, with more than one-third of participants meeting their primary goal. No participants discontinued due to adverse events or lack of improvement.

**Winston et al. 2023** [34] (ROBINS-I overall: Serious)

This prospective safety-focused study enrolled 113 patients across three cohort studies at a single institution (59 in the upper limb study, 43 in the tibial nerve study, 11 in the bilateral obturator study). CNL was performed on 277 nerves (99 mixed motor–sensory). One patient developed a local skin infection and two patients had bruising or swelling, all resolving within one month. Nine patients reported nerve pain or dysaesthesia: two involving purely motor nerve targets, seven involving mixed sensorimotor nerve targets. Overall, 96.75% of nerve treatments produced no pain or dysaesthesia beyond the procedural period. Symptoms of three patients persisted to 3 months; one had hypoaesthesia at 6 months. Seven patients withdrew (mean 5.4 months), four died from unrelated causes; none of these 11 reported adverse events. One patient received botulinum toxin for cramps. This study was formally dedicated to safety; spasticity outcome data were not reported.

#### 3.4.3. Case Series

**Pick, Dye & Fleming 2025** [39] (JBI: Moderate)

Eight patients with diverse neurological diagnoses (age range 25–75 years) underwent CNL at an Oxford spasticity clinic—the first published CNL for spasticity case series originating from the United Kingdom. All patients underwent prior DNBs with local anaesthetic to confirm suitability. Assessment included GAS, MAS, ArmA, LegA, PRISM, and patient satisfaction questionnaires at baseline and at regular intervals over 9–12 months. All patients achieved at least one treatment goal, with effects sustained beyond 6 months. ArmA/LegA measures showed several significant improvements, particularly in passive function. PRISM demonstrated notable reduction in the negative impact of spasticity across all domains, stabilised at 6 months. Three patients reported hypoaesthesia: forearm hypoaesthesia after musculocutaneous nerve branch treatment (resolved at 6 months), thigh-to-leg hypoaesthesia after obturator nerve treatment (resolved at 12 months), and little finger hypoaesthesia after ulnar nerve treatment (persistent at 12 months). Post-procedural pain was the most common adverse effect, resolving in all affected patients within 3 months.

**Winston et al. 2019** [23] (JBI: Low)

The seminal paper proposing the Canadian CNL for spasticity protocol described 3 patients with mixed diagnoses treated in upper and lower limbs, with follow-up for up to 17 months, along with a narrative literature review identifying only two prior publications on CNL for spasticity (Kim & Ferrante 1998 [22], and one unpublished abstract). A proof-of-concept abstract evaluating 19 patients with musculocutaneous nerve CNL (iovera° device) reported 79% responders (MAS reduction ≥1 point) at 4 weeks. This publication has primarily historical significance as the methodological foundation for subsequent CANOSC studies.

#### 3.4.4. Case Reports: Synthesis by Outcome Domain

**A. Spasticity (MAS/MTS)**

A consistent pattern of immediate MAS reduction was documented across all 10 case reports, with persistence ranging from weeks to beyond 12 months. Key findings include: MAS reduction sustained at 15 months in an ambulatory MS patient following femoral nerve branch CNL, with re-treatment performed at that time point (Boissonnault et al. 2024 [47]); immediate MAS and ROM improvement, sustained at 8 months in a 14-year-old with cerebral palsy and Haemophilia A, without haemorrhagic complications using Factor VIII prophylaxis (Mumby et al. 2025 [40]); caregiver-reported improvements in upper body dressing (CareQ score 4→3), washing (4→2), transfers (3→2), and wheelchair positioning at 8.5 months following bilateral lateral pectoral nerve CNL in a Gross Motor Function Classification System (GMFCS) 5 child (Scobie & Winston 2021 [41]); and in one case, limited MAS response despite ROM improvement was documented: in a patient with C4 incomplete quadriplegia and severe hip osteoarthritis, MAS was not significantly reduced, likely attributable to severe joint restrictions, joint pain, and concurrent pressure ulcers rather than procedural failure (Schatz et al. 2024 [45]).

**B. Range of Motion**

Passive ROM (Tardieu V1) was the outcome with the highest frequency and consistency of improvement. Chantraine et al. 2026 [35] uniquely corroborated ROM improvements with quantitative laboratory gait analysis and fine-wire EMG: the GDI rose from 69 to 80 (surpassing the published minimal detectable difference of ≥5 points [49]), and the Gillette Gait Index decreased by more than 55% (surpassing the minimal clinically important difference of ≥30% [50]). Peak swing-phase knee flexion improved from 32.8° at baseline to 53.5° at 6-month follow-up, normalising the stiff-knee gait pattern. This case additionally demonstrated, for the first time, the compatibility and synergistic interaction between CNL and an implanted common peroneal functional electrical stimulation (FES) system: activating the FES device reduced the Gillette Gait Index by a further 23–40%, suggesting that debulking antagonist tone enlarges the biomechanical window within which a neuroprosthesis can operate.

**C. Pain**

Pain associated with spasticity was the second-most frequently reported outcome. Di Lorenzo et al. 2026 [36] documented complete resolution of movement-related pain (Visual Analogue Scale [VAS] during movement: from 7–8 to 3–4 at one-month follow-up) following bilateral proximal CNL of lateral pectoral, thoracodorsal, and musculocutaneous nerves combined with distal BoNT-A injections in a patient with hereditary spastic paraplegia-like neurodegenerative disorder. This case additionally illustrates the novel combined proximal CNL–distal BoNT-A paradigm aimed at reducing pathological proximal synergies while preserving residual distal voluntary function, with the patient reporting preserved and subjectively improved dexterity for computer keyboard use.

**D. Functional Outcomes and Quality of Life**

Formal functional outcome assessment was limited almost exclusively to Pick et al. 2025 [39] and Chantraine et al. 2026 [35]. The sole quality of life and cost-effectiveness evaluation was restricted to Hughes et al. 2025 [42] (n = 1), which documented 15-fold annual cost savings compared to BoNT-A in the Canadian healthcare context. This figure derives from a single case in a specific reimbursement setting and cannot be generalised.

#### 3.4.5. Special Populations

The exploratory nature of the literature has led to progressive extension of CNL to populations with specific clinical needs not previously studied:

**Haemophilia A:** Mumby et al. 2025 [40] provides the first report of CNL in a patient with elevated haemorrhagic risk secondary to Haemophilia A, using pre-procedural Factor VIII prophylaxis, with a favourable outcome. This constitutes an evidence level of a single case report (Very Low quality).

**Paediatric population:** Scobie & Winston 2021 [41] reports caregiver-perspective outcomes in a 15-year-old with GMFCS 5 cerebral palsy. CNL has reportedly been performed in children as young as 3 years and in nonagenarians [11,41]; no systematic evidence is available for the paediatric age group.

**Pregnancy:** A case study from the CANOSC group describes a pregnant patient with MS who underwent tibial nerve CNL as BoNT-A was contraindicated during pregnancy [23]. Foetal safety cannot be evaluated from anecdotal-level evidence.

**Quadriplegia with severe orthopaedic deformity:** Schatz et al. 2024 [45] documents the first CNL application in a patient with C4 incomplete quadriplegia and severe hip osteoarthritis, demonstrating immediate ROM gains and patient-reported improvements in independence including independent showering, fall prevention, and cessation of hip flexor spasms.

**Post-stroke with implanted FES:** Chantraine et al. 2026 [35] is the first study to document CNL in a patient with an implanted common peroneal FES device, demonstrating compatibility and synergistic interaction.

#### 3.4.6. Aggregated Safety Profile

The overall rate of minor local adverse events (infection, bruising) in the largest structured dataset is approximately 2–3%, comparable to that of ultrasound-guided percutaneous procedures in general. The differential risk of sensory disturbance—approximately 10-fold higher for mixed sensorimotor targets (7.1%) than for purely motor targets (1.1%) per treated nerve [34]—has direct clinical implications for nerve target selection and patient-specific informed consent. This differential is of particular relevance in upper extremity practice, where sensory function of the hand is of primary functional importance: treatment of mixed nerve trunks in the upper limb (e.g., musculocutaneous nerve at trunk level, median nerve above the elbow) must be preceded by explicit patient-specific risk–benefit discussion, with DNB pre-screening used to verify clinical indication.Aggregated safety profile are resumed in Table 9.

**Important caveat:** Safety data originate almost exclusively from a single clinical group (CANOSC/VIHA). The absence of standardised adverse-event collection frameworks (e.g., Common Terminology Criteria for Adverse Events [CTCAE]) in the majority of included studies limits comparability. The long-term safety profile (>12 months, multiple treatment cycles on the same nerve) remains unsystematically documented.

#### 3.4.7. Ongoing Trials and Research Landscape

Since its introduction in 2018, CNL for spasticity has been adopted in multiple centres across Canada, the United States, Luxembourg, the United Kingdom, France, Denmark, and Spain [11]. This clinical diffusion, substantially preceding the availability of high-quality controlled evidence, is a recurring pattern in procedural technology adoption and imposes particular responsibility on the scientific community.

Multiple controlled trials addressing the primary evidence gaps are currently in the treatment or recruitment phase. Studies in France comparing neurectomy versus cryoneurolysis, and botulinum toxin versus cryoneurolysis in the UK and Luxembourg, and a large multicentre sham-controlled study in the USA are all entering the treatment phase.

In the United Kingdom, the pilot randomised controlled trial registered as **ISRCTN50433077**—Cryoneurolysis to treat pain in the context of spasticity—directly compares CNL with botulinum toxin injection. The NHS Foundation Trust has been offering this treatment routinely since January 2024. This pilot study aims to improve understanding of the potential effectiveness of the treatment and its potential side effects when compared with a more commonly used treatment (Botulinum Toxin) [2].

In Luxembourg, the **spastiCRYO-LL** trial—Study on the efficacy of selected peripheral nerves cryoneurolysis to treat the functional problems, disability burden, and pain caused by spasticity due to central nervous system pathology—received ethics committee approval in April 2024 and is supported by the Luxembourgish Ministry of Health and Social Security. Its protocol has generated the first quantitative gait-laboratory evidence for CNL, as published by Chantraine et al. (2026) [35] within this review.

In Canada, **NCT06958289** (Cryoneurolysis for Spasticity Treatment: Long-term Clinical Outcomes and Mechanisms in the Central Nervous System) is a single-centre mechanistic pilot study at Parkwood Institute (London, Ontario), targeting 25 patients with stroke. Its primary objective is not efficacy but neuroplasticity characterisation via transcranial magnetic stimulation (TMS), functional MRI, and functional near-infrared spectroscopy (fNIRS), testing the hypothesis that axonotmesis-induced peripheral silencing triggers central neuroplastic reorganisation that may account for tone reductions persisting beyond nerve regeneration.

## 4. Discussion

### 4.1. Summary of Principal Findings

This systematic scoping review identifies and synthesises the entirety of available evidence on percutaneous CNL as a treatment for focal and multifocal spasticity. The evidence base is composed of studies of predominantly low or very low methodological quality (GRADE ⊕◯◯◯ for efficacy; ⊕⊕◯◯ for safety), with no published RCTs. In the absence of published sham-controlled or comparator-group data, no definitive causal conclusions regarding CNL efficacy can be drawn.

Within these constraints, the available data support three observations: (1) across observational studies, case series, and case reports—predominantly from a single clinical centre—ultrasound-guided percutaneous CNL demonstrates a reproducible pattern of improvements in passive ROM (Tardieu V1), MAS score reduction, and pain relief, sustained up to 12–15 months; (2) the safety profile in available structured data is favourable, with total adverse event rates for purely motor nerve treatments below 2% and a clinically actionable differential risk for mixed sensorimotor targets; (3) CNL has been employed predominantly in patients with spasticity refractory to or plateaued on BoNT-A, positioning it clinically as a second-line or complementary intervention.

It must be stated unambiguously that even these three observations—describing pre-post patterns within uncontrolled single-arm studies rather than treatment effects—are insufficient to support routine clinical adoption outside of carefully designed controlled trials or prospective registries with systematic outcome collection. The GRADE Very Low (⊕◯◯◯) certainty rating assigned to all efficacy outcomes is not a minor qualification: by GRADE convention, it formally indicates that the true effect of CNL may be substantially different from—or in a direction opposite to—the observed pre-post changes reported in the included studies.

### 4.2. CNL in the Context of Current Treatment Options

Botulinum toxin type A remains the gold standard for focal spasticity, supported by Level A evidence [12,16]. Three structural limitations—a median 12–16-week efficacy window, regulatory dose ceilings, and progressive tachyphylaxis—create the clinical rationale for alternative approaches [16,17]. No study in this review directly compared CNL and BoNT-A in a controlled fashion. The only available comparative datum is the anecdotal 15-fold annual cost saving from a single Canadian case report [42], which cannot be generalised to other healthcare systems.

Chemical neurolysis (phenol/alcohol) provides 3–6 months of tone reduction but carries risks of painful dysaesthesia (10–30%) and myofibrosis [18]. The mechanistic distinction is critical: CNL at suitable temperatures produces reversible Wallerian degeneration (Sunderland Grade II axonotmesis) with preserved connective tissue sheaths and effectively eliminates neuroma risk [24]; phenol/alcohol induce irreversible neurotmesis with permanent dysaesthesia risk. No direct controlled comparison of CNL and chemical neurolysis for spasticity has been published.

Intrathecal baclofen is effective for severe generalised spasticity through a distinct supraspinal/spinal mechanism [19]. CNL is a focal peripheral nerve technique; its clinical niche is focal or multifocal spasticity, not generalised spasticity.

Selective surgical neurotomy achieves greater and more sustained spasticity reduction than BoNT-A [20] but is irreversible and carries surgical risk. The reversibility of CNL constitutes its principal theoretical advantage over neurotomy at the cost of limited duration and the need for re-treatment. Head-to-head comparative trials of CNL versus selective neurotomy are currently in the treatment phase in France [51].

The proximal CNL–distal BoNT-A combination paradigm, first systematically described in Di Lorenzo et al. 2026 [36], represents a conceptually novel dual-modulation strategy in which CNL reduces pathological proximal synergies while BoNT-A is reserved for distal fine-tuning, preserving essential hand function. This approach deserves prospective evaluation.

A clinically relevant extension of the CNL evidence base concerns its potential applicability in post-traumatic spasticity and complex upper extremity injuries. Traumatic brain injury (TBI) and spinal cord injury (SCI) are well-established aetiologies of upper motor neuron spasticity in the reviewed literature (Hashemi et al. 2025 [37]; Kim & Ferrante 1998 [22]; Schatz et al. 2024 [45]). In the broader context of interventional pain medicine and peripheral nerve surgery, cryoanalgesia has been employed for decades to manage post-operative and post-traumatic pain through temporary peripheral nerve blockade, with a well-characterised mechanism of reversible Wallerian degeneration [21,52]. In upper extremity traumatology specifically, spasticity co-existing with structural musculoskeletal injuries—such as spastic elbow flexor contracture complicating TBI-associated heterotopic ossification, or spastic hand deformity in post-traumatic tetraparesis—represents a therapeutically challenging target. In these complex injury contexts, CNL may serve a dual role: as a tone-modulating intervention and as a reversible pre-surgical functional evaluation tool, providing the multidisciplinary team with information regarding anticipated functional gain under simulated low-tone conditions prior to irreversible surgical commitment. This potential application in traumatology is mechanistically supported by the established reversibility of Sunderland Grade II axonotmesis [24] but lacks dedicated prospective clinical evidence. Studies examining CNL efficacy and safety in post-traumatic populations with complex musculoskeletal injury patterns are warranted and represent a clinically important extension of the current research agenda.

#### Specific Implications for Hand and Upper Extremity Surgery

The evidence reviewed has direct implications for clinicians involved in the surgical and non-surgical management of upper limb spasticity, and generates three clinically specific observations.

**CNL as a pre-surgical functional evaluation tool.** Selective surgical neurotomy achieves greater and more sustained spasticity reduction than BoNT-A [20] but is irreversible, and once performed, precludes functional re-evaluation under different tone conditions. For the hand and upper extremity surgeon, the reversibility of CNL constitutes a potential asset beyond its role as a standalone treatment: the temporary axonotmesis it produces [24] allows functional assessment during the period of peripheral nerve silencing, providing the multidisciplinary team with pre-decisional information about anticipated functional gain under simulated low-tone conditions. This role is mechanistically analogous to, but of substantially longer duration than, a diagnostic nerve block with local anaesthetic, and may assist in candidate selection and timing for irreversible surgical procedures. This potential application warrants prospective investigation in dedicated upper extremity surgery cohorts.

**The DNB protocol and surgical candidate selection.** The diagnostic nerve block protocol systematically employed by the CANOSC group [34] and adopted in Pick et al. 2025 [39] has direct relevance to surgical planning: a positive DNB response identifies candidates likely to benefit from peripheral nerve intervention, while also providing the surgeon with pre-operative functional information without committing to an irreversible procedure. The predictive value of the DNB response for CNL outcome has not been systematically analysed in any included study (Table 10, Gap #4), representing a priority for centres combining surgical and non-surgical spasticity management.

**The proximal CNL–distal BoNT-A paradigm in upper extremity practice.** The proximal CNL–distal BoNT-A combination paradigm first described in Di Lorenzo et al. 2026 [36] is of particular interest in the upper extremity context: CNL of proximal motor branches (pectoral, thoracodorsal, musculocutaneous nerves) reduces pathological proximal flexor synergies while BoNT-A is reserved for distal fine-tuning, preserving essential hand function. This dual-modulation strategy corresponds directly to the clinical goal pursued in multidisciplinary upper extremity spasticity surgery—reducing proximal tone while protecting residual distal voluntary function—and deserves prospective evaluation in controlled studies involving hand surgery centres.

### 4.3. Clinical Implications and Patient Selection

Based on the available evidence classified as GRADE ⊕◯◯◯ for efficacy and ⊕⊕◯◯ for safety, the following conditional clinical indications can be formulated, consistent with weak GRADE recommendations:

CNL **should not** be offered as a first-line alternative to BoNT-A in treatment-naive patients, in the absence of comparative evidence of adequate quality.

CNL **may be considered**—following multidisciplinary specialist assessment and adequate patient information—in focal or multifocal spasticity refractory to or plateaued on BoNT-A; in cases where BoNT-A is contraindicated; or as a complementary approach within combined treatment strategies.

DNB with local anaesthetic is an essential prerequisite for both candidate selection and informed consent based on the simulated procedural outcome.

Treatment of mixed sensorimotor nerves must be performed with explicit caution and patient-specific informed consent regarding the elevated sensory disturbance risk (7.1% per treated nerve vs. 1.1% for purely motor targets [34]).

Prescribing and procedural responsibilities for CNL rest with physician specialists with demonstrated competence in ultrasound-guided peripheral nerve procedures—typically in physical medicine and rehabilitation, pain medicine, or rehabilitation neurology—operating within a multidisciplinary spasticity programme in which the hand and upper extremity surgeon plays an active and central role in patient selection, functional outcome interpretation, and irreversible surgical decision-making

CNL has been included as a non-pharmacological option in the 2024 AAPM&R consensus statement on spasticity management [11], representing growing institutional recognition of the technique.

### 4.4. Evidence Gaps

Evidence gaps are resumed in Table 10.

**Table 10:** Critical evidence gaps in the CNL for the spasticity literature.

	Research Question (PICO)	Gap Identified	Study Design Required
1	Is CNL superior to BoNT-A in treatment-naive patients with focal spasticity?	No head-to-head RCT	Double-blind sham-controlled RCT
2	What is the duration of CNL effect by nerve target type?	Systematic follow-up >12 months absent	Prospective multicentre study, 24-month follow-up
3	Does CNL modify the natural history of spasticity or its progression towards contracture?	No study has evaluated this outcome	RCT or prospective cohort with structural outcomes
4	What is the predictive value of the diagnostic nerve block for CNL outcome?	No study has analysed DNB-CNL response correlation	Prospective observational study with predictive analysis
5	What is the safety profile of CNL across multiple cycles (>3) on the same nerve?	No systematic data on repeat treatments	Prospective safety cohort
6	Is CNL cost-effective vs. BoNT-A in national European healthcare systems?	Single case report datum in a specific Canadian context	Formal health economic analysis based on RCT
7	Which patient characteristics predict CNL response?	No systematic subgroup analysis available	Regression analysis on expanded dataset
8	What is the safety and efficacy of CNL in paediatric CP populations?	Single case report (Scobie & Winston 2021) [41]	RCT or paediatric cohort study
9	What is the mechanism of nerve regeneration post-CNL and its functional implications?	Mechanistic data in humans absent	Advanced neuroimaging (MR neurography, serial nerve conduction studies)
10	Is CNL safe and effective in patients with coagulation disorders?	Single case report (Mumby et al. 2025) [40]	Cohort study in patients with coagulopathies
11	Can CNL serve as a pre-surgical functional evaluation tool to inform selective neurotomy candidate selection in upper extremity spasticity?	No prospective study has examined this role	Prospective comparative study in multidisciplinary upper extremity centres

### 4.5. Limitations of This Review

**Publication bias:** The limited number of studies and their near-exclusive origin from a single clinical group increase the probability of positive publication bias.

**Single-institution concentration:** Results may not be reproducible at other centres or by operators with different training; potential salami-slicing of shared datasets inflates the apparent consistency of the evidence base.

**Cohort overlap:** The confirmed overlap between Hashemi et al. 2025 [37] and Winston et al. 2023 [34] (sharing NCT04670783) may inflate the perceived size of the evidence base.

**Non-standardised outcome battery:** Heterogeneity of outcome measures precluded quantitative synthesis.

**Absence of prospective protocol registration:** This represents a methodological limitation of the present review.

**Terminology heterogeneity:** CNL for spasticity is a field with non-standardised terminology (cryoneurolysis, cryoneurotomy, cryoneuroablation); some relevant studies using non-conventional terminology may not have been captured despite the broad search strategy.

Evidence gaps are resumed in Table 10.

## 5. Conclusions

### 5.1. Principal Findings

The overall certainty of evidence, assessed using the GRADE framework, is Very Low (⊕◯◯◯) for all efficacy outcomes—including spasticity reduction (MAS), range of motion improvement, pain relief, functional outcomes, and quality of life—and Low (⊕⊕◯◯) for safety. These ratings reflect: the complete absence of published randomised controlled trial data; a Critical-to-Serious overall risk of bias in the two largest primary clinical studies (ROBINS-I: Critical for Hashemi et al. 2025 [37]; Serious for Winston et al. 2023 [34]); single-institution concentration of primary evidence (≥69% of primary clinical studies from one research group); confirmed patient cohort overlap between the two largest primary studies; financial conflicts of interest with the primary device manufacturer identified in ≥48% of included studies (≥12/25); and systematic outcome assessment without assessor blinding.

This systematic scoping review, covering all published literature through February 2026, synthesises the entirety of available evidence on percutaneous CNL for spasticity across all aetiological groups and anatomical regions. Twenty-five studies met pre-specified eligibility criteria. No RCTs were identified

Within these critically constrained evidentiary boundaries—which render the following observations hypothesis-generating rather than confirmatory, and which preclude any clinical recommendation based on the direction or magnitude of observed effect—and with the explicit caveat that pre-post improvements cannot be causally attributed to treatment in the absence of a controlled comparator, the literature consistently describes improvements in passive ROM (Tardieu V1), MAS scores, and pain relief following ultrasound-guided percutaneous CNL. In the largest prospective observational cohort (Hashemi et al. 2025 [37], n = 59, 12-month follow-up), statistically significant improvements were documented across multiple upper limb regions—including shoulder, elbow, wrist, and finger/thumb targets—in a population with spasticity refractory to BoNT-A. In the prospective safety study (Winston et al. 2023 [34], n = 113, 277 nerves), 96.75% of nerve treatments produced no sensory adverse effects beyond the procedural period; the differential risk of dysaesthesia between mixed sensorimotor and purely motor nerve targets (7.1% vs. 1.1% per treated nerve) is an actionable clinical signal, with particular relevance in the upper extremity where hand sensory function is of primary importance.

These findings must be interpreted with explicit acknowledgement of five structural limitations: absence of any randomised or controlled comparison; single-institution concentration of primary evidence (≥69% of primary clinical studies from one research group); confirmed cohort overlap between the two largest primary studies; financial conflicts of interest with the primary device manufacturer identified in ≥48% of included studies; and systematic outcome assessment without assessor blinding. Current conclusions must therefore be framed as hypothesis-generating rather than practice-changing, pending the availability of results from ongoing controlled trials.

### 5.2. Clinical Positioning

Based on the available evidence, CNL may be considered a clinically justifiable second-line or complementary intervention—in centres with demonstrated expertise in ultrasound-guided peripheral nerve procedures and within a multidisciplinary spasticity programme—for patients presenting with: (1) focal or multifocal spasticity refractory to or plateaued on BoNT-A; (2) spasticity that has reached the regulatory dose ceiling or is complicated by tachyphylaxis; (3) clinical conditions in which BoNT-A is contraindicated; (4) complex upper limb spasticity in which a combined proximal CNL–distal BoNT-A strategy may preserve essential distal voluntary function while reducing pathological proximal synergies.

In the specific context of hand and upper extremity surgery, CNL may additionally represent a clinically coherent pre-surgical functional evaluation instrument: the reversible axonotmesis it produces [24] allows functional assessment under simulated low-tone conditions prior to an irreversible surgical decision, a role mechanistically supported by the established value of the DNB protocol in candidate selection [34] but of substantially longer duration and functional clarity. This potential application—which positions CNL within the therapeutic continuum between reversible pharmacological intervention and irreversible surgical neurotomy—warrants prospective investigation in dedicated upper extremity surgery cohorts.

CNL is **not** currently supported by evidence of sufficient quality to recommend its use as a first-line alternative to BoNT-A in treatment-naive patients. DNB pre-screening is an essential prerequisite. Treatment of mixed sensorimotor nerve trunks requires specific informed consent. All prescribing and procedural responsibilities rest exclusively with the treating specialist physician.

### 5.3. Research Priorities

The most critical evidence gap is the complete absence of published RCT data. Multiple ongoing trials—a large multicentre sham-controlled trial in the United States, comparative trials of CNL versus BoNT-A in the United Kingdom and Luxembourg, and comparative trials of CNL versus selective neurotomy in France—are expected to provide substantially higher-quality evidence. These trials must be conducted with adequate statistical power, formally blinded outcome assessors, validated primary outcomes beyond the MAS, minimum 12-month follow-up, and pre-specified subgroup analyses by aetiology, anatomical region, age, and prior BoNT-A exposure.

Six additional research priorities are identified: (1) development of a core outcome set for CNL trials in spasticity to enable cross-study comparison; (2) multicentre studies to assess reproducibility and generalisability beyond the single-institution evidence base; (3) long-term follow-up beyond 12 months and characterisation of safety across multiple treatment cycles; (4) dedicated studies in underrepresented populations (children with cerebral palsy, patients with coagulation disorders, pregnant women with spasticity); (5) mechanistic studies characterising Wallerian degeneration and reinnervation kinetics in humans following clinical-grade CNL; and (6) formal health economic analyses in multiple healthcare systems.

A seventh research priority, specific to the context of hand and upper extremity surgery, is identified: prospective investigation of CNL as a pre-surgical functional evaluation tool in patients being considered for selective neurotomy or other irreversible upper extremity procedures, examining whether reversible peripheral nerve silencing improves surgical candidate selection, outcome prediction, and patient-reported satisfaction with surgical outcomes.

### 5.4. Original Contribution

To our knowledge, this represents the first scoping review to: map the entirety of published evidence on CNL for spasticity across all aetiological groups and anatomical regions; systematically appraise methodological quality using design-appropriate tools (JBI Critical Appraisal Checklists, ROBINS-I, AMSTAR-2); apply the GRADE framework to the evidence base as a whole; explicitly characterise the pervasive financial conflict of interest landscape; document confirmed cohort overlap between the two largest primary studies; identify device heterogeneity across the corpus as an additional source of technical variability not previously addressed in narrative reviews; and contextualise the available evidence within the clinical decision-making framework relevant to hand and upper extremity surgery specialists.

### 5.5. Statement for Clinical Practice and Health Policy

Clinical adoption of CNL for spasticity has proceeded, internationally, substantially faster than the generation of high-quality controlled evidence.

**Clinicians** should inform patients that CNL represents a promising but early-stage intervention whose superiority over established treatments has not been demonstrated in controlled conditions, and that available evidence derives predominantly from a single research group with documented financial ties to the primary device manufacturer.

**Researchers** must ensure that ongoing and future trials are conducted with strict methodological rigour, including assessor blinding, sham controls, standardised outcomes, and a priori registration; independent funding sources should be sought whenever possible.

**Healthcare decision-makers** should condition reimbursement decisions on evidence from adequately powered, independent, controlled trials, while recognising the legitimate clinical need that has driven current adoption.

The field of CNL for spasticity is at an inflection point: the mechanistic rationale is sound, the safety profile in available studies is encouraging, and the unmet clinical need is substantial for millions of patients living with disabling spasticity worldwide. For hand and upper extremity specialists in particular, CNL represents a mechanistically coherent addition to the therapeutic continuum between reversible pharmacological intervention and irreversible surgical neurotomy—occupying a clinical niche that has thus far remained inadequately addressed. Realising the potential of this intervention requires the generation of high-quality, independent, methodologically rigorous evidence—the standard that every emerging treatment in evidence-based medicine must ultimately meet.

## Figures and Tables

**Table 1:** Quality assessment of the study.

Study Design	n	Tool Applied
Case reports	10	JBI Critical Appraisal Checklist for Case Reports (8 items)
Case series	3	JBI Critical Appraisal Checklist for Case Series (10 items) [30]
Observational studies (single-arm before-after)	2	ROBINS-I (7 domains; adapted for single-arm design) [31]
Systematic/scoping reviews	2	AMSTAR-2 (16 items) [32]
Narrative reviews, technical notes, device review	8	JBI Textual Evidence appraisal

**Table 2:** Patient Cohort Independence Status and Registry Cross-Reference across primary clinical studies (n = 16).

Study	Registry ID	N Enrolled (Total)	Nerve Targets	Independence Status
Hashemi et al. 2025 [37]	NCT04670783	59 (upper limb only)	Upper limb	Confirmed subset of Winston et al. 2023; NOT an independent sample
Winston et al. 2023 [34]	NCT04670783 + 2 additional	113 total (59 UL + 43 tibial + 11 obturator)	Upper + lower limb	Contains Hashemi et al. 2025 cohort; net non-overlapping contribution: 54 patients (43 tibial + 11 obturator)
Pick et al. 2025 [39]	Not pre-registered	8	Upper + lower limb	Independent (Oxford, UK)
Winston et al. 2019 [23]	Not registered	3 (cases) + 19 (abstract)	Mixed	Potentially overlapping with later CANOSC studies; unconfirmable without individual-level data
Case reports (n = 10) [22,35,40,41,42,43,44,45,46,47]	N/A	1 each (n = 10 total)	Mixed	Independent (geographically and institutionally diverse)
Net independent primary clinical sample (estimated)		≈155–170 (upper bound ≈200–215 prior to overlap correction)		After removal of confirmed NCT04670783 cohort overlap

Abbreviations: UL = upper limb; N/A = not applicable. Footnote: Confirmed overlap is based on Winston et al. 2023 [34] explicit statement that 59 participants were enrolled in the upper limb study (NCT04670783), subsequently reported as the primary efficacy cohort of Hashemi et al. 2025 [37]. Additive combination of patient totals across these two studies constitutes double-counting and must be explicitly avoided in evidence interpretation and sample size estimation. The net non-overlapping patient contribution of Winston et al. 2023 [34] to the aggregate clinical evidence base is 54 individuals (tibial nerve cohort: n = 43; bilateral obturator cohort: n = 11).

**Table 3:** Devices for cryoneurolisis.

Study	Device	Cryogen	Temperature	Manufacturer
CANOSC studies (majority)	iovera°	N_2_O	−88 °C	Pacira BioSciences, Inc.
Hashemi et al. 2025 [37]	iovera° and Lloyd SL 2000	N_2_O/CO_2_	−88 °C/−60 °C	Pacira/Neurostat Spembly Medical, London, UK
Scobie & Winston 2021 [41]	Lloyd SL 2000	CO_2_	−60 °C	Neurostat Spembly Medical
Chantraine et al. 2026 [35]	Metrum Cryoflex (N_2_O-based)	N_2_O	−30 to −40 °C tissue	Metrum Cryoflex, Warsaw, Poland
Di Lorenzo et al. 2026 [36]	Cryo-S Painless	CO_2_	−70 °C (device spec)	Metrum Cryoflex, Łomianki, Poland

**Table 4:** Characteristics of primary clinical studies included in the scoping review (n = 16).

Category	Duration	Studies
Short-term	≤3 months	Di Lorenzo et al. 2026 [36] (1–2 months); Herzog et al. 2023 [43] (8 weeks)
Medium-term	3–6 months	David et al. 2024 [44] (4 months); Mumby et al. 2025 [40] (8 months); Schatz et al. 2024 [45] (9 months); MacRae et al. 2023 [46] (12 weeks + re-treatment at 6 months)
Long-term	>6 months	Scobie & Winston 2021 [41] (9 months); Chantraine et al. 2026 [35] (6 months from CNL-1); Hughes et al. 2025 [42] (11 months); Hashemi et al. 2025 [37] (12 months); Winston et al. 2023 [34] (minimum 3 months); Boissonnault et al. 2024 [47] (15 months)

**Table 5:** JBI Quality Assessment—case reports (n = 10).

Study	Author, Year	Item 1: Demographics	Item 2: Timeline	Item 3: Clinical Condition	Item 4: Diagnostics	Item 5: Intervention	Item 6: Post-Intervention	Item 7: Adverse Events	Item 8: Takeaway Lessons	Overall Quality
2	Hughes 2025 [42]	Y	Y	Y	Y	Y	Y	Y	Y	High
3	Mumby 2025 [40]	Y	Y	Y	Y	Y	Y	Y	Y	High
8	MacRae 2023 [46]	Y	Y	Y	Y	Y	Y	Y	Y	High
6	Boissonnault 2024 [47]	U	Y	Y	Y	Y	Y	Y	Y	Moderate
1	Chantraine 2026 [35]	U	Y	Y	Y	Y	Y	Y	Y	Moderate
7	Herzog 2023 [43]	U	N	Y	Y	Y	Y	Y	Y	Moderate
5	David 2024 [44]	U	N	Y	Y	Y	Y	Y	Y	Low–Moderate
9	Scobie 2021 [41]	U	N	Y	Y	Y	Y	Y	Y	Low
4	Schatz 2024 [45]	U	N	Y	U	Y	Y	Y	Y	Low
10	Kim 1998 [22]	N	N	U	Y	U	Y	N	Y	Low

Y = Yes; N = No; U = Unclear/Not reported. Overall quality: High ≥ 7 Y; Moderate = 5–6 Y; Low ≤ 4 Y.

**Table 6:** JBI Quality Assessment—case series (n = 3).Y = Yes (criterion clearly met and explicitly documented in the study); N = No (criterion absent or explicitly not fulfilled); U = Unclear/Not reported (criterion cannot be assessed due to insufficient reporting; treated as a methodological limitation equivalent to non-fulfilment for quality rating purposes). Overall quality rating: High ≥ 7 items rated Y; Moderate 5–6 items rated Y; Low ≤ 4 items rated Y, per JBI Critical Appraisal Checklist for Case Series (10 items).

Item	Winston 2019 [23]	Pick 2025 [39]	Guynn 2025 [38]
1. Inclusion criteria defined	U	Y	U
2. Standard, reliable measurement	Y	Y	U
3. Valid methods for outcome identification	Y	Y	U
4. Consecutive inclusion	N	U	U
5. Complete inclusion	N	U	U
6. Clear reporting of demographics	N	Y	U
7. Clear reporting of clinical information	Y	Y	U
8. Outcomes/follow-up clearly reported	Y	Y	Y
9. Appropriate statistical analysis	N	N	N
10. Adverse events identified and described	Y	Y	Y
Overall quality	Low	Moderate	Low–Critically Low

**Table 7:** ROBINS-I Assessment—observational studies (n = 2).

ROBINS-I Domain	Hashemi et al. 2025 [37]	Winston et al. 2023 [34]
D1: Confounding	Critical	Serious
D2: Selection of participants	Serious	Serious
D3: Classification of interventions	Low	Low
D4: Deviations from intended interventions	Moderate	Moderate
D5: Missing data	Serious	Moderate
D6: Measurement of outcomes	Serious	Serious
D7: Selection of reported results	Critical	Serious
overall judgement	critical	serious

**Table 8:** GRADE Evidence Profile—Key Outcome Domains. GRADE certainty of evidence symbols: Each outcome domain was assessed using the GRADE (Grading of Recommendations Assessment, Development and Evaluation) framework. Certainty is represented by a combination of filled (⊕) and open (◯) circles across four levels: ⊕⊕◯◯ = Low (further research very likely to have an important impact); ⊕◯◯◯ = Very Low (any estimate of effect is highly uncertain). The starting certainty for non-randomised observational evidence is Low (⊕⊕◯◯); further downgrading was applied for risk of bias, indirectness, imprecision, and publication bias across all efficacy domains. Safety outcomes were downgraded one level rather than two, reflecting the partial availability of prospectively collected adverse-event data. Abbreviations: MAS = Modified Ashworth Scale; GAS = Goal Attainment Scaling; ArmA = Arm Activity measure; CR = case report; CoI = conflict of interest; AE = adverse event; N = number of included studies.

Outcome Domain	No. Studies	Certainty	Main Reasons for Downgrading
Spasticity reduction (MAS)	16	⊕◯◯◯ Very Low	Risk of bias (critical–serious); indirectness (single-arm, no control); imprecision (small N); publication bias
Range of motion improvement	14	⊕◯◯◯ Very Low	As above
Pain reduction	8	⊕◯◯◯ Very Low	As above + outcome heterogeneity
Safety/adverse events	12 (2 structured + 10 CR)	⊕⊕◯◯ Low	Risk of bias (no systematic AE collection); imprecision
Functional outcomes (GAS, ArmA)	1	⊕◯◯◯ Very Low	Single study, small N, CoI
Quality of life/cost-effectiveness	1	⊕◯◯◯ Very Low	Single case report

**Table 9:** Aggregated Safety Profile—data from studies with Structured Adverse-Event Reporting. N/A: Not applicable.

Type of AE	n (Events)	Source	Course
Local skin infection	1/113 (0.9%)	Winston et al. 2023 [34]	Resolved within 1 month
Bruising/swelling	2/113 (1.8%)	Winston et al. 2023 [34]	Resolved within 1 month
Nerve pain/dysaesthesia—motor nerves	2/178 purely motor nerves (1.1%)	Winston et al. 2023 [34]	Mostly <3 months
Nerve pain/dysaesthesia—mixed sensorimotor nerves	7/99 mixed nerves (7.1%)	Winston et al. 2023 [34]	3 persistent at 3 months; 1 at 6 months
Cramp pain (treated with BoNT-A)	1/113 (0.9%)	Winston et al. 2023 [34]	Not reported
Forearm hypoaesthesia	1/8 (12.5%)	Pick et al. 2025 [39]	Resolved at 6 months
Thigh-to-leg hypoaesthesia	1/8 (12.5%)	Pick et al. 2025 [39]	Resolved at 12 months
Little finger hypoaesthesia	1/8 (12.5%)	Pick et al. 2025 [39]	Persistent at 12 months
Immediate procedural pain	≥4/10 case reports	Multiple	Transient
Haemorrhagic complications (Haemophilia A)	0/1	Mumby et al. 2025 [40]	N/A
Deaths	4/113	Winston et al. 2023 [34]	All unrelated to procedure

## Data Availability

No new data were created or analysed in this study. Data sharing is not applicable to this article.

## Supplementary Materials

The following supporting information can be downloaded at: https://www.mdpi.com/article/10.3390/jcm15093541/s1, Table S1: Preferred Reporting Items for Systematic reviews and Meta-Analyses extension for Scoping Reviews (PRISMA-ScR) Checklist [27].

## References

[1] Lance J.W. (1980). The control of muscle tone, reflexes, and movement: Robert Wartenberg Lecture. Neurology.

[2] Pandyan A.D., Gregoric M., Barnes M.P., Wood D., Van Wijck F., Burridge J., Hermens H., Johnson G. (2005). Spasticity: Clinical perceptions, neurological realities and meaningful measurement. Disabil. Rehabil..

[3] Watkins C.L., Leathley M.J., Gregson J.M., Moore A.P., Smith T.L., Sharma A.K. (2002). Prevalence of spasticity post stroke. Clin. Rehabil..

[4] Rizzo M.A., Hadjimichael O.C., Preiningerova J., Vollmer T.L. (2004). Prevalence and treatment of spasticity reported by multiple sclerosis patients. Mult. Scler..

[5] Skold C., Levi R., Seiger A. (1999). Spasticity after traumatic spinal cord injury: Nature, severity, and location. Arch. Phys. Med. Rehabil..

[6] Nielsen J.B., Crone C., Hultborn H. (2007). The spinal pathophysiology of spasticity—from a basic science point of view. Acta Physiol..

[7] Wissel J., Verrier M., Simpson D.M., Charles D., Guinto P., Papapetropoulos S., Sunnerhagen K.S. (2015). Post-stroke spasticity: Predictors of early development and considerations for therapeutic intervention. PM&R.

[8] Brainin M., Norrving B., Sunnerhagen K.S., Goldstein L.B., Cramer S.C., Donnan G.A., Duncan P.W., Francisco G., Good D., Graham G. (2011). Poststroke chronic disease management: Towards improved identification and interventions for poststroke spasticity-related complications. Int. J. Stroke.

[9] Ward A.B., Roberts G., Warner J., Gillard S. (2005). Cost-effectiveness of botulinum toxin type A in the treatment of post-stroke spasticity. J. Rehabil. Med..

[10] Gracies J.M. (2005). Pathophysiology of spastic paresis. I: Paresis and soft tissue changes. Muscle Nerve.

[11] Khan F., Amatya B., Bensmail D., Yelnik A. (2024). Pharmacological and non-pharmacological management of spasticity: An umbrella review of systematic reviews. Pharmaceuticals.

[12] Simpson D.M., Hallett M., Ashman E.J., Comella C.L., Green M.W., Gronseth G.S., Armstrong M.J., Gloss D., Potrebic S., Jankovic J. (2016). Practice guideline update summary: Botulinum neurotoxin for the treatment of blepharospasm, cervical dystonia, adult spasticity, and headache. Neurology.

[13] Deltombe T., Lejeune T., Gustin T. (2019). Botulinum toxin type A or selective neurotomy for treating focal spastic muscle overactivity?. Ann. Phys. Rehabil. Med..

[14] Rubenstein J., Harvey A.W., Vincent D., Winston P. (2021). Cryoneurotomy to reduce spasticity and improve range of motion in spastic flexed elbow: A visual vignette. Am. J. Phys. Med. Rehabil..

[15] Ali I., Vincent D., Winston P. (2025). Cryoneurolysis for the forearm in spasticity management: A video gallery. Am. J. Phys. Med. Rehabil..

[16] Francisco G.E., Balbert A., Bavikatte G., Bensmail D., Carda S., Deltombe T., Draulans N., Escaldi S., Gross R., Jacinto J. (2021). A practical guide to optimizing the benefits of post-stroke spasticity interventions with botulinum toxin A: An international group consensus. J. Rehabil. Med..

[17] Turner-Stokes L., Fheodoroff K., Jacinto J., Maisonobe P., Lysandropoulos A. (2013). Results from the Upper Limb International Spasticity Study-II (ULIS-II): A large, international, prospective cohort study investigating practice and goal attainment following treatment with botulinum toxin A in real-life clinical management. BMJ Open.

[18] Li S., Winston P., Mas M.F. (2024). Spasticity treatment beyond botulinum toxins. Phys. Med. Rehabil. Clin. N. Am..

[19] Plassat R., Perrouin Verbe B., Menei P., Menegalli D., Mathé J.F., Richard I. (2004). Treatment of spasticity with intrathecal baclofen administration: Long-term follow-up, review of 40 patients. Spinal Cord.

[20] Deltombe T., Bleyenheuft C., Gustin T. (2015). Comparison between tibial nerve block with anaesthetics and neurotomy in hemiplegic adults with spastic equinovarus foot. Ann. Phys. Rehabil. Med..

[21] Trescot A.M. (2003). Cryoanalgesia in interventional pain management. Pain Physician.

[22] Kim P.S., Ferrante F.M. (1998). Cryoanalgesia: A novel treatment for hip adductor spasticity and obturator neuralgia. Anesthesiology.

[23] Winston P., Mills P.B., Reebye R., Vincent D. (2019). Cryoneurotomy as a percutaneous mini-invasive therapy for the treatment of the spastic limb: Case presentation, review of the literature, and proposed approach for use. Arch. Rehabil. Res. Clin. Transl..

[24] Hsu M., Stevenson F.F. (2015). Wallerian degeneration and recovery of motor nerves after multiple focused cold therapies. Muscle Nerve.

[25] Arksey H., O’Malley L. (2005). Scoping studies: Towards a methodological framework. Int. J. Soc. Res. Methodol..

[26] Levac D., Colquhoun H., O’Brien K.K. (2010). Scoping studies: Advancing the methodology. Implement. Sci..

[27] Tricco A.C., Lillie E., Zarin W., O’Brien K.K., Colquhoun H., Levac D., Moher D., Peters M.D.J., Horsley T., Weeks L. (2018). PRISMA extension for scoping reviews (PRISMA-ScR): Checklist and explanation. Ann. Intern. Med..

[28] Page M.J., McKenzie J.E., Bossuyt P.M., Boutron I., Hoffmann T.C., Mulrow C.D., Shamseer L., Tetzlaff J.M., Akl E.A., Brennan S.E. (2021). The PRISMA 2020 statement: An updated guideline for reporting systematic reviews. BMJ.

[29] Aromataris E., Lockwood C., Porritt K., Pilla B., Jordan Z. (2024). JBI Manual for Evidence Synthesis.

[30] Munn Z., Barker T.H., Moola S., Tufanaru C., Stern C., McArthur A., Stephenson M., Aromataris E. (2020). Methodological quality of case series studies: An introduction to the JBI critical appraisal tool. JBI Evid. Synth..

[31] Sterne J.A.C., Hernán M.A., Reeves B.C., Savović J., Berkman N.D., Viswanathan M., Henry D., Altman D.G., Ansari M.T., Boutron I. (2016). ROBINS-I: A tool for assessing risk of bias in non-randomised studies of interventions. BMJ.

[32] Shea B.J., Reeves B.C., Wells G., Thuku M., Hamel C., Moran J., Tugwell P., Welch V., Kristjansson E., Henry D.A. (2017). AMSTAR 2: A critical appraisal tool for systematic reviews that include randomised or non-randomised studies of healthcare interventions, or both. BMJ.

[33] Guyatt G.H., Oxman A.D., Vist G.E., Kunz R., Falck-Ytter Y., Alonso-Coello P., Schünemann H.J. (2008). GRADE: An emerging consensus on rating quality of evidence and strength of recommendations. BMJ.

[34] Winston P., MacRae F., Rajapakshe S., Morrissey I., Boissonnault È., Vincent D., Hashemi M. (2023). Analysis of adverse effects of cryoneurolysis for the treatment of spasticity. Am. J. Phys. Med. Rehabil..

[35] Chantraine F., Pereira J.A., Schreiber C., Classen T., Areno G., Dierick F. (2026). Targeted and sequential cryoneurolysis improves gait after botulinum-toxin unresponsiveness in post-stroke spasticity: A laboratory-verified case. Neurol. Int..

[36] Di Lorenzo L., De Meo B., Forte A.M., Forte F., Palmieri V., Pirraglia N., D’Avanzo C. (2026). Exploratory use of proximal cryoneurolysis and distal botulinum toxin type A for upper-limb spasticity: A case report with scoping review. Toxins.

[37] Hashemi M., MacRae F., Boissonnault È., Vincent D., Song J., Shi S., Winston P. (2025). Measuring the efficacy of percutaneous cryoneurolysis in participants with refractory or plateaued shoulder, elbow, wrist, or finger spasticity. Am. J. Phys. Med. Rehabil..

[38] Guynn C.C. (2025). Percutaneous cryoneurolysis as a dynamic treatment for spasticity: A case series. Arch. Rehabil. Res. Clin. Transl..

[39] Pick A., Dye R., Fleming M.K. (2025). Cryoneurolysis: A novel treatment for management of spasticity. Presentation of a case series. Adv. Rehabil. Sci. Pract..

[40] Mumby G., Schatz L., Claridge E., Hashemi M., Winston P. (2025). A case report of cryoneurolysis with factor VIII administration for cerebral palsy-related spasticity in a patient with hemophilia A. Adv. Rehabil. Sci. Pract..

[41] Scobie J., Winston P. (2021). Case report: Perspective of a caregiver on functional outcomes following bilateral lateral pectoral nerve cryoneurotomy to treat spasticity in a pediatric patient with cerebral palsy. Front. Rehabil. Sci..

[42] Hughes A., Hashemi M., Schatz L., Gatenby D., Winston P. (2025). Cryoneurolysis for managing spasticity in multiple sclerosis: A case report demonstrating sustained functional gains and cost-effectiveness. Arch. Rehabil. Res. Clin. Transl..

[43] Herzog S., David R., Speirs A., Hashemi M., Winston P. (2023). A case report illustrating the combined use of cryoneurolysis and percutaneous needle tenotomy in the treatment of longstanding spastic shoulder contractures after stroke. Arch. Rehabil. Res. Clin. Transl..

[44] David R., Hashemi M., Schatz L., Winston P. (2024). Multisite treatment with percutaneous cryoneurolysis for upper and lower limb in long-standing post-stroke spasticity. Eur. J. Phys. Rehabil. Med..

[45] Schatz L., Herzog S., Hashemi M., Winston P. (2024). Cryoneurolysis and quadriplegia: A case report on pain and severe spasticity management. Arch. Rehabil. Res. Clin. Transl..

[46] MacRae F., Speirs A., Bursuc A., Hashemi M., Winston P. (2023). A case report of cryoneurolysis for dorsal foot pain and toe clawing in a patient with multiple sclerosis. Arch. Rehabil. Res. Clin. Transl..

[47] Boissonnault È., MacRae F., Hashemi M., Bursuc A., Winston P. (2024). Cryoneurolysis of the femoral nerve for focal spasticity in an ambulatory patient. Arch. Rehabil. Res. Clin. Transl..

[48] Muradia S., Prasad R. (2026). Indications, trends, and outcomes of nerve blocks for spasticity management: A systematic review. NeuroRehabilitation.

[49] Schwartz M.H., Rozumalski A. (2008). The gait deviation index: A new comprehensive index of gait pathology. Gait Posture.

[50] Cretual A., Bervet K., Ballaz L. (2010). Gillette Gait Index in adults. Gait Posture.

[51] Winston P., Vincent D. (2024). Cryoneurolysis as a novel treatment for spasticity, associated pain and presumed contracture. Adv. Rehabil. Sci. Pract..

[52] Ilfeld B.M., Preciado J., Trescot A.M. (2016). Novel cryoneurolysis device for the treatment of sensory and motor peripheral nerves. Expert Rev. Med. Devices.

